# Supplementary figures and images for: Assessing hypoxic respiratory failure in mechanically ventilated neonates: A comparative study of oxygen saturation index and oxygenation index
Source: PLoS One. 2024 May 30;19(5):e0304278. doi: 10.1371/journal.pone.0304278 (PMC11139266; doi:10.1371/journal.pone.0304278)

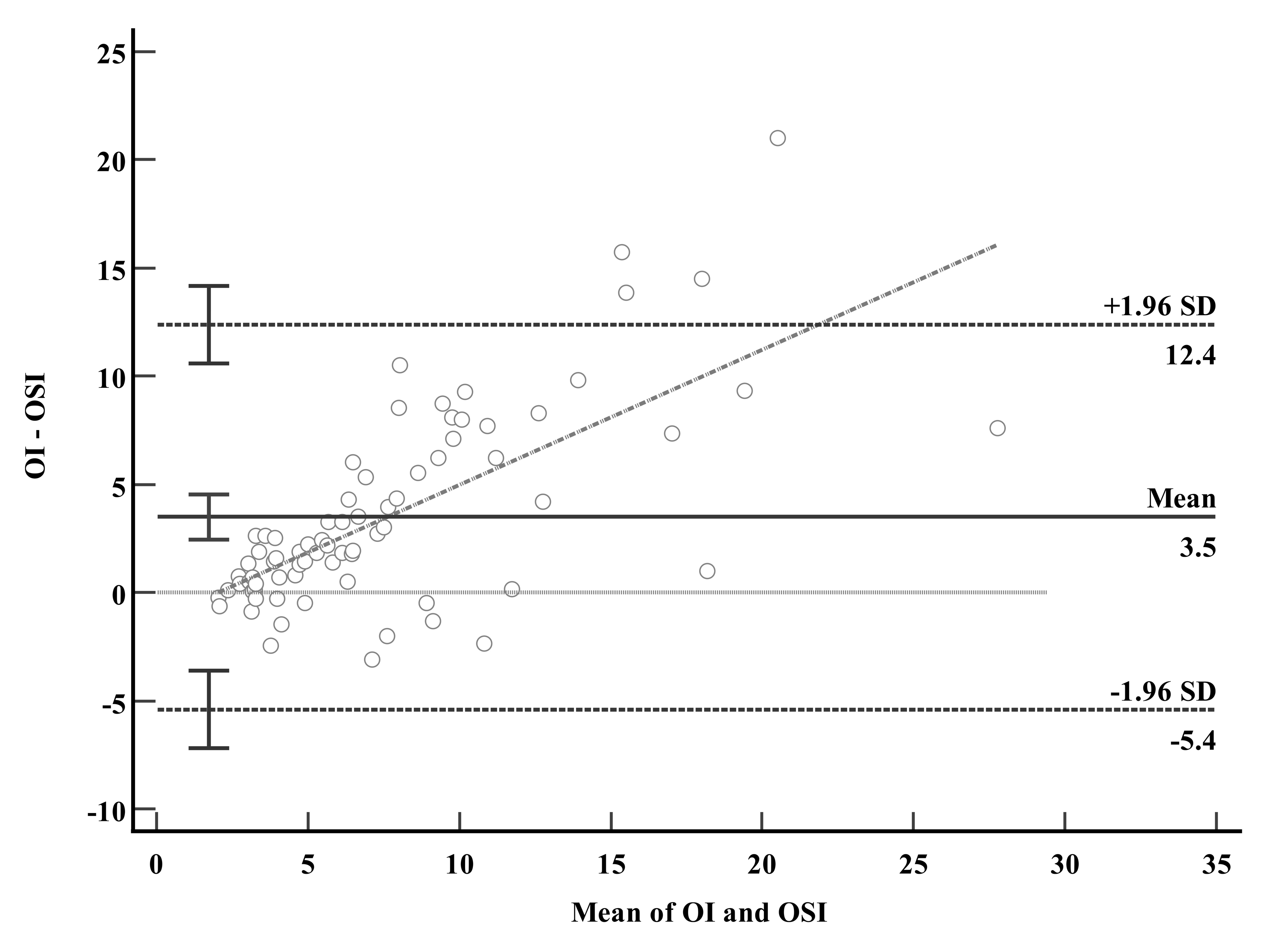

Supplement: S1 Fig — The diagonal line represents the slope of the relationship indicating a significant bias, p < 0.0001. (TIF) [file pone.0304278.s001.tif]

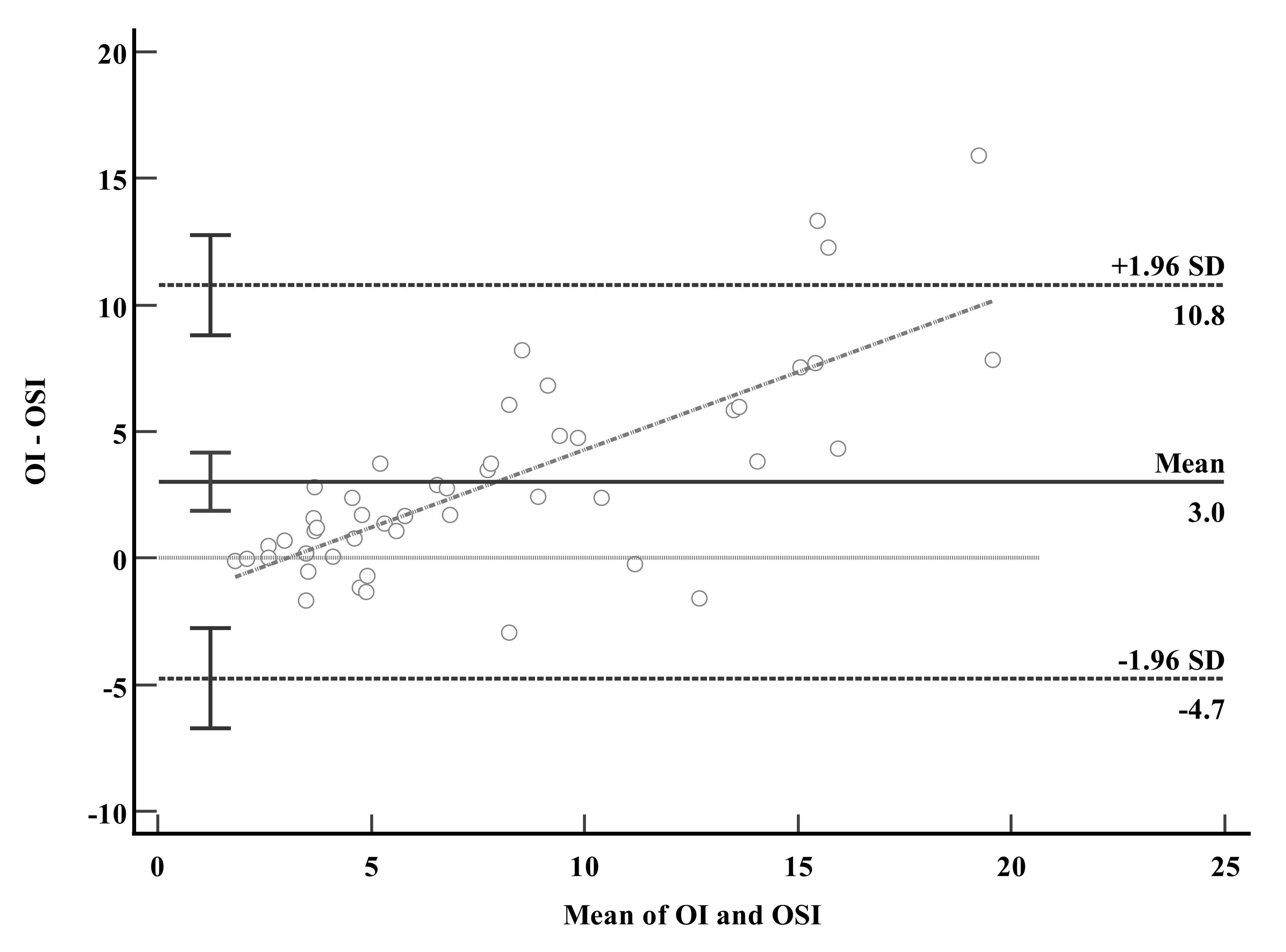

Supplement: S2 Fig — The diagonal line represents the slope of the relationship indicating a significant bias, p < 0.0001. (TIF) [file pone.0304278.s002.tif]
